# A shotgun antisense approach to the identification of novel essential genes in *Pseudomonas aeruginosa*

**DOI:** 10.1186/1471-2180-14-24

**Published:** 2014-02-05

**Authors:** Ruggero Rusmini, Davide Vecchietti, Raffaella Macchi, Faustino Vidal-Aroca, Giovanni Bertoni

**Affiliations:** 1 Department of Life Sciences, Università degli Studi di Milano, via Celoria 26, 20133 Milan, Italy; 2 Alcon Italia S.p.A., viale Richard 1/B, 20143 Milan, Italy

## Abstract

**Background:**

Antibiotics in current use target a surprisingly small number of cellular functions: cell wall, DNA, RNA, and protein biosynthesis. Targeting of novel essential pathways is expected to play an important role in the discovery of new antibacterial agents against bacterial pathogens, such as *Pseudomonas aeruginosa*, that are difficult to control because of their ability to develop resistance, often multiple, to all current classes of clinical antibiotics.

**Results:**

We aimed to identify novel essential genes in *P. aeruginosa* by shotgun antisense screening. This technique was developed in *Staphylococcus aureus* and, following a period of limited success in Gram-negative bacteria, has recently been used effectively in *Escherichia coli*. To also target low expressed essential genes, we included some variant steps that were expected to overcome the non-stringent regulation of the promoter carried by the expression vector used for the shotgun antisense libraries. Our antisense screenings identified 33 growth-impairing single-locus genomic inserts that allowed us to generate a list of 28 “essential-for-growth” genes: five were “classical” essential genes involved in DNA replication, transcription, translation, and cell division; seven were already reported as essential in other bacteria; and 16 were “novel” essential genes with no homologs reported to have an essential role in other bacterial species. Interestingly, the essential genes in our panel were suggested to take part in a broader range of cellular functions than those currently targeted by extant antibiotics, namely protein secretion, biosynthesis of cofactors, prosthetic groups and carriers, energy metabolism, central intermediary metabolism, transport of small molecules, translation, post-translational modification, non-ribosomal peptide synthesis, lipopolysaccharide synthesis/modification, and transcription regulation. This study also identified 43 growth-impairing inserts carrying multiple loci targeting 105 genes, of which 25 have homologs reported as essential in other bacteria. Finally, four multigenic growth-impairing inserts belonged to operons that have never been reported to play an essential role.

**Conclusions:**

For the first time in *P. aeruginosa*, we applied regulated antisense RNA expression and showed the feasibility of this technology for the identification of novel essential genes.

## Background

*Pseudomonas aeruginosa* is a highly adaptable bacterium that thrives in a broad range of ecological niches. In addition, it can infect hosts as diverse as plants, nematodes, and mammals. In humans, it is an important opportunistic pathogen in compromised individuals, such as patients with cystic fibrosis, severe burns, or impaired immunity [1,2]. *P. aeruginosa* is difficult to control because of its ability to develop resistance, often multiple, to all current classes of clinical antibiotics [3-5]. The discovery of novel essential genes or pathways that have not yet been targeted by clinical antibiotics can underlie the development of alternative effective antibacterials to overcome existing mechanisms of resistance. Whole-genome transposon-mutagenesis (TM) followed by identification of insertion sites is one of the most practical and frequently used experimental approaches to screen for essential bacterial genes [6-8]. Genome-wide surveys of essential genes in *P. aeruginosa* have been accomplished by saturating TM through a “negative” approach [9,10], specifically, by identifying non-essential genomic regions by transposon insertion and deducing that non-inserted genome stretches are essential. However, this approach can suffer from some systematic biases that generate both false positives and negatives [7]. For example, even if comprehensive insertion libraries are produced, it is inevitable that some genes, especially the shortest ones, could elude insertion and be spuriously annotated as essential, while transposon insertions that occur at gene ends and do not fully inactivate the function could lead to genes being incorrectly classified as non-essential. To filter errors resulting from these intrinsic biases in the “negative” TM approach, a statistical framework has recently been developed and tested in *P. aeuginosa* PAO1 and *Francisella tularensis novicida*[7] TM datasets. Some drawbacks of the “negative” TM approach were overcome by using growth-conditional TM, which allows identification of essential genes by transcriptionally fusing them to an outward-facing inducible promoter located at one end of the transposon [11]. However, conditional TM can also be affected by systematic biases, deriving, for example, from transposon tools endowed with outward-facing promoters that are not strictly regulated in non-inducing conditions, resulting in a basal level of promoter expression. In fact, promoter leakage under non-inducing conditions would not completely switch off the gene downstream of the insertion site, significantly increasing the false-negative identification rate. The TM tools applicable for use with *P. aeruginosa*[12] are based on elements used for tightly regulated gene expression in *E. coli*, and are expected to not be completely switched off in non-inducing conditions when used “out-of-context”. For these reasons, we set out to screen novel essential genes of *P. aeruginosa* using a method other than TM. To this end, we selected shotgun antisense RNA identification of essential genes, a technique that was developed a decade ago in *Staphylococcus aureus*[13,14]. This technique originally only showed limited success in Gram-negative bacteria [15,16], but has recently been used effectively in *E. coli*[17]. In this approach, essential genes are identified after shotgun-cloned genomic fragments are conditionally expressed. The fragments are screened to identify those whose expression impairs growth [18]. The genes targeted by antisense RNA are identified by DNA sequencing of the growth-impairing fragments. This study shows for the first time the feasibility of the antisense technology in *P. aeruginosa* for identifying novel essential genes. Moreover, we included some modifications to the original strategy that could have broadened the functional class variety of the identified essential genes in respect to a recent report in *E. coli*[17].

## Results

### *Ad hoc* procedure to screen for essential *P. aeruginosa* genes by antisense RNA effects

**Figure 1:**
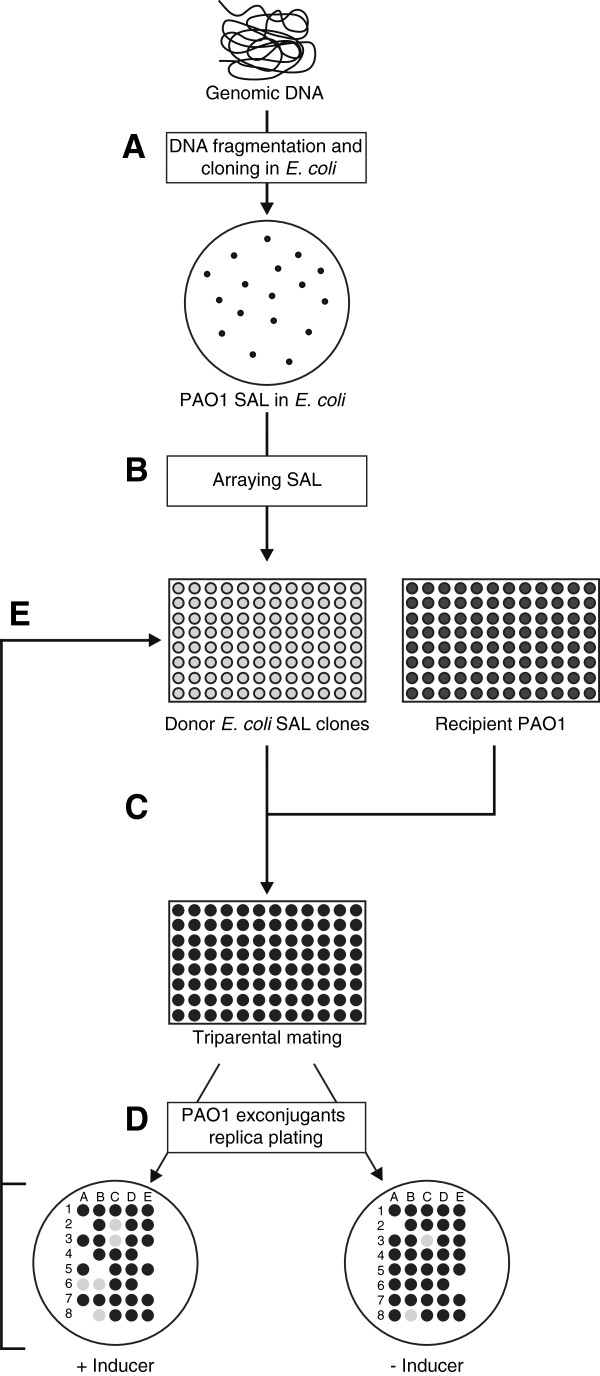
**Construction and screening of PAO1 SALs. (A)** Genomic DNA was isolated from *P. aeruginosa* PAO1 and nebulized to obtain sheared fragments of 200–800 bp. After treatment with exonuclease BAL-31 and Klenow polymerase, the genomic DNA fragments were cloned into the *E. coli* strain JM109, downstream of the arabinose-inducible promoter P_BAD_ of the pHERD20T vector. **(B)***E. coli* transformants, representing the PAO1 shotgun antisense library (SAL), were arrayed in 96-well microplates and **(C)** mated with *P. aeruginosa* PAO1 in the presence of a helper strain (triparental mating). **(D)** SAL recipient PAO1 exconjugants were selected by spotting on PIA plates supplemented with Cb both in the absence and in the presence of the P_BAD_ inducer arabinose. Recipient PAO1 exconjugant spots were inspected for growth defects following 24 h of incubation at 37°C. **(E)** The identity of the genomic fragments eliciting growth defects (lethal effects, indicated by a lack of a spot: only with inducer, e.g. clones A4, A8, B5, and E4, and with and without an inducer, e.g. clones A2 and E6; growth impairment, indicated as gray spots: only with an inducer, e.g. clones C2, A6, and B6, and with and without an inducer, e.g. C3 and B8) was determined by sequencing the inserts in the corresponding clones of *E. coli* SAL.

According to the scheme for antisense-mediated identification of essential genes established in *S. aureus*[13,14], the shotgun genomic libraries generated *in vitro* are directly introduced into the original host by transformation, and selected in permissive conditions, i.e., with the promoter vector in an off state, to allow the clones carrying inserts targeting essential genes to survive. However, basal vector promoter activity could be sufficient to elicit silencing effects against genes transcribed at low levels. This effect may introduce a bias in the subsequent conditional screening, favoring the identification of highly transcribed essential genes (e.g., tRNAs, tRNA synthetases, ribosomal proteins, translation factors, components of the transcription machinery). Cells transformed using constructs targeting essential genes expressed at low levels will fail to form a colony in the permissive conditions. The high basal *P*_*trc*_ activity was suggested to cause the identification of a relevant proportion of highly expressed essential genes in a recent antisense screening of *E. coli*[17]. A not entirely negligible basal activity is frequent in the commonly used expression system tools, especially when they are used outside the source organism. This is the case in the *P*_*BAD*_ promoter-based systems, like those selected for this study, which have been used for tightly regulated gene expression in *E. coli*, and for efficient arabinose-induced overexpression in other hosts. However, outside of the *E. coli* regulatory context, for instance in *Burkholderia pseudomallei*[19] and *P. aeruginosa* (Bertoni *et al.*, unpublished), these systems can display, also in the presence of glucose, a basal level of activity. To avoid missing the identification of low expressed essential genes owing to out-of-context use of the *P*_*BAD*_ promoter, we set out to generate *P. aeruginosa* genomic shotgun libraries in *E. coli* first, and to then array and challenge them by conjugative transfer into *P. aeruginosa* (Figure 1). Moreover, this strategy assures a larger sized shotgun library because of the higher transformation efficiency of *E. coli* compared with *P. aeruginosa.* To test the robustness of this approach, we checked the false-positive rate due to failure of vector mating transfer and assessed that it was negligible.

### Construction of arrayed shotgun genomic libraries of *P. aeruginosa*

Genomic DNA was purified from *P. aeruginosa* PAO1 and then mechanically sheared to generate DNA fragments in a size range spanning 200–800 bp (Additional file 1: Figure S1A). In pilot experiments, following treatment with exonuclease BAL-31 and Klenow polymerase, the 200–800 bp DNA fragments were cloned into *E. coli* downstream of the arabinose-inducible promoter, *P*_*BAD*_, of the broad host-range vector pVI533EH. Approximately 800 transformant clones were then arrayed in 96-well microplates. Analysis of cloning efficiency by PCR indicated that about 30% of transformant *E. coli* colonies carried a PAO1 genomic insert. To generate shotgun antisense libraries (SALs) with a lower background of clones carrying an empty vector, we selected the broad host-range vector pHERD-20 T, which facilitates the identification of clones carrying an insert based on blue/white screening. We obtained a 7:3 ratio between dark blue (absence of an insert) and white-light blue (potential presence of an insert) colonies, with 95% of white-light blue colonies carrying an insert with the expected average size (Additional file 1: Figure S1B). Thus, the probability of selecting a clone with an insert (Additional file 1: Figure S1C) increased from about 30% to 95% using pHERD-20 T. A pHERD-20 T-based SAL library was constructed by arraying approximately 10,000 white-light blue transformant clones in 96-well microplates.

### Screenings of SALs for growth-impairing inserts

The genomic inserts of both pVI533EH- and pHERD-20 T-based SALs were screened for their ability to impair PAO1 growth, supposedly by antisense transcription effects, by mating transfer of SALs from *E. coli* to PAO1 (Figure 1C), and then replica plating of exconjugants on *Pseudomonas* Isolation Agar (PIA) supplemented with carbenicillin (Cb), both in the absence and presence of the *P*_*BAD*_ inducer arabinose (Figure 1D). Recipient PAO1 exconjugant spots were inspected for growth defects following 24 h of incubation at 37°C. Insert-induced impairment ranged from growth defect to arrest, which could be displayed in some cases even in the absence of arabinose (Additional file 1: Figure S1C). This suggested that basal insert expression in PAO1, a regulatory context for *P*_*BAD*_ that is not as restrictive as *E. coli*, was sufficient to produce deleterious effects on growth. These screenings resulted in the identification of five and 71 growth-impairing inserts in the pVI533EH- and pHERD-20 T-based SALs, respectively. These 76 inserts, recovered in the corresponding *E. coli* donor clones (Figure 1E), were subjected to sequence analysis, and their features are listed in Additional file 2: Table S2.

### Analysis of the growth-impairing inserts

Bioinformatic analysis of the DNA sequences obtained indicated that 33 of the 76 positive clones (44%) contained single intragenic fragments. Of these, 20 (26% of the positive clones) were in antisense orientation. As listed in Table 1, some of these fragments derived from conserved genes involved in DNA replication, transcription, and translation, such as *dnaG*, *rpoC*, *rpoB*, *infB*, and *rbfA*, which can be considered “classical” essential genes. Fragments derived from *rpoC*, *rpoB*, *infB*, and *rbfA* were antisense oriented. Two different fragments were derived from *dnaG,* one antisense and the other sense oriented. As previously suggested [13], it is likely that sense-oriented intragenic fragments can act as dominant-negative interfering sequences. Alternatively, we suggest that transcription noise in the vector backbone from the other side of *P*_*BAD*_ could produce sufficient amounts of insert antisense transcripts to silence the target essential gene. One insert targeted PA3820 (*secF*), which was previously shown to play an essential role in several bacterial species [20]. Six intragenic fragments derived from PA4669 (*ipk*), PA2951 (*etfA*), PA3687 (*ppc*), PA3758 (*nagA*), PA1183 (*dctA*), and PA1805 (*ppiD*), which are homologous to genes previously shown to be essential in a limited number of bacterial species [20].

**Table 1:** ***Pseudomonas aeruginosa *** **PAO1 genes targeted by growth-impairing inserts including a single *locus***

**Insert name**^**a**^	**Insert-included *locus***	**Gene name and product annotation**^**b**^	**Function class**^**b**^	**Orthologous proteins in DEG**^**c**^
**S2F1**	PA0577	*dnaG* - DNA primase (2)	DNA replication, recombination, modification and repair	Bs, Sa, Ec, Hi, Sp, Mt, Fn, Ab, Mp, Se, Cc, Ss
S3D3				
**S11F7**	PA4269	*rpoC* - DNA-directed RNA polymerase beta chain (2)	Transcription, RNA processing and degradation	Bs, Sa, Ec, Mg, Sp, Mt, St, Fn, Ab, Mp, Se, Ss, Pg
**B1**	PA4270	*rpoB* - DNA-directed RNA polymerase beta chain (2)	Transcription, RNA processing and degradation	Bs, Sa, Vc, Ec, Hi, Mg, Mt, St, Fn, Ab, Mp, Pa, Se, Cc, Ss, Pg, Bt
**S6A10**	PA4744	*infB* - translation initiation factor IF-2 (2)	Translation, post-translational modification, degradation	Bs, Sa, Vc, Ec, Mg, Mt, St, Fn Ab, Mp, Pa, Se, Cc, Pg, Bt
**S2F6**	PA4743	*rbfA* - ribosome-binding factor A (2)	Translation, post-translational modification, degradation	Sa, Vc, Mg, Hp, Fn, Ab, Se
S6E7	PA3820	*secF* - secretion protein SecF (2)	Protein secretion/export apparatus	Ec, Hi, St, Fn, Ab, Pa, Se
**S5A10**	PA1709^d^	*popD* - Translocator outer membrane protein (1)	Protein secretion/export apparatus	
**S11B13**	PA4669	*ipk* - isopentenyl monophosphate kinase(2)	Biosynthesis of cofactors, prosthetic groups and carriers	Vc, Se, Pa
**G2**	PA2951	*etfA* - electron transfer flavoprotein alpha-subunit (2)	Energy metabolism	Ab, Cc
**H2**				
S10F8	PA5186	probable iron-containing alcohol dehydrogenase (3)	Energy metabolism	
F1	PA1554	*ccoN1*\ *fixN* \ Cytochrome c oxidase, cbb3-type, CcoN subunit (1)	Energy metabolism	
S11G10	PA3687	*ppc* - phosphoenolpyruvate carboxylase (2)	Energy metabolism	Hi
S86C	PA3758	*nagA* - probable N-acetylglucosamine-6-phosphate deacetylase (3)	Central intermediary metabolism	Hi, Mt
**E5**	PA1183	*dctA* - C4-dicarboxylate transport protein (2)	Transport of small molecules	Ab
**S11C9**	PA3382	*phnE* - phosphonate transport protein PhnE (2)	Transport of small molecules	
S4E6	PA4903	*vanK* - probable major facilitator superfamily (MFS) transporter (3)	Transport of small molecules	
**B3**	PA5548	probable major facilitator superfamily (MFS) transporter (3)	Transport of small molecules	
S4H12				
A5	PA1590	*braB* - branched chain amino acid transporter (1)	Transport of small molecules	
**S3D4**	PA1805	*ppiD* - peptidyl-prolyl cis-trans isomerase D - Rotamase D (2)	Translation, post-translational modification, degradation	Cc, Bs
**S9G5**	PA2402	Probable non-ribosomal peptide synthetase (3)	Putative enzymes	
S5D4				
S5A4	PA5238	probable O-antigen acetylase (3)	Membrane proteins, Cell wall/LPS/capsule	
**S5G6**				
S4B10	PA3433	*ywbI* - probable transcriptional regulator (3)	Transcriptional regulators	
**S5A1**	PA2220	*oprR* - probable transcriptional regulator (3)	Transcriptional regulators	
**M4G6**	PA2873^e^	*tgpA* - transglutaminase protein A TgpA (1)	Adaptation, Protection, Membrane proteins	
**S10A3**	PA0307	hypothetical protein (4)	Hypothetical, unclassified, unknown	
**S841F**	PA4926	conserved hypothetical protein (4)	Hypothetical, unclassified, unknown	
**S9A9**	PA0262	conserved hypothetical protein (4)	Hypothetical, unclassified, unknown	
F2	PA5264	hypothetical protein (4)	Hypothetical, unclassified, unknown	

^a^Inserts with antisense orientation are in bold.

^b^Annotations according to the Pseudomonas Genome Database (http://www.pseudomonas.com) [27]. Numbers inside parenthesis indicate the classes of product name confidence. Class1: Function experimentally demonstrated in *P. aeruginosa*; Class 2: Function of highly similar gene experimentally demonstrated in another organism; Class 3: Function proposed based on presence of conserved amino acid motif, structural feature or limited sequence similarity to an experimentally studied gene. Class 4: Homologs of previously reported genes of unknown function, or no similarity to any previously reported sequences.

^c^DEG: Database of Essential Genes (DEG 7.0) (http://www.essentialgene.org) [20]. For each hit, the orthologue proteins, extracted from the Ortholuge DB [30], that were found in DEG are indicated with the abbreviation of the harboring bacterial species. Bacterial species: Ab (*Acinetobacter baylyi*), Bs (*Bacillus subtilis*), Bt (*Bacteroides thetaiotaomicron*), Cc (*Caulobacter crescentus*), Ec (*Escherichia coli*), Fn (*Francisella novicida*), Hi (*Haemophilus influenzae*), Hp (*Helicobacter pylori)*, Mt (*Mycobacterium tuberculosis*), Mp (*Mycoplasma pulmonis*), Mg (*Mycoplasma genitalium*), Pg (*Porphyromonas gingivalis*), Pa (*Pseudomonas aeruginosa*), Se (*Salmonella enterica*), St (*Salmonella typhimurium*), Sp (*Streptococcus pneumoniae*), Ss (*Streptococcus sanguinis*), Sa (S*taphylococcus aureus*), Vc (*Vibrio cholerae*).

^d^Previous reports [24-26] did not mention growth defects associated to deletion of *popD* gene.

^e^Hit not present in DEG whose essentiality was experimentally demonstrated in *P. aeruginosa*[21].

The other inserts shown in Table 1 are derived from 16 genes with no homologs annotated as essential [20]. We recently validated the essential role of one of these hits, TgpA (PA2873), in *P. aeruginosa* by insertional and conditional mutagenesis [21]. In addition, the critical role of PA1554 (*ccoN1*) in the aerobic growth of *P. aeruginosa* PAO1 was reported previously [22].

The remaining positive clones contained fragments including multiple loci and targeted a total of 103 genes (Additional file 3: Table S3). Nineteen of these multigenic fragments included 25 genes with homologs described as essential in other bacterial species [20]. The rest of the multigenic fragments carried genes with no evidence of an essential role. Interestingly, four multigenic inserts included gene sequences belonging to a single operon (Table 2).

**Table 2:** PAO1 growth-impairing inserts including loci belonging to a single operon

**Insert name**^**a**^	**Operon *loci***^**b**^	**Gene name and product annotation**^**c**^	**Function class**^**c**^	**Species containing orthologs in DEG**^**d**^
**E6**	**PA1037**	*yicG* - conserved hypothetical protein (4)	Hypothetical, unclassified, unknown	
	**PA1038**	hypothetical protein (4)		
	PA1039	*ychJ* - hypotetical protein (4)		
	PA1040	hypothetical protein (4)		
S9B6a	**PA1089**	conserved hypothetical protein (4)	Hypothetical, unclassified, unknown	
	**PA1090**	conserved hypothetical protein (4)		
	PA1088	hypothetical protein (4)		
**S9B6b**	**PA0393**	*proC* - pyrroline-5-carboxylate reductase (1)	Amino acid biosynthesis and metabolism	*E. coli*, *M. tuberculosis*, *A. baylyi*
	**PA0392**	*yggT* - conserved hypothetical protein (4)	Hypothetical, unclassified, unknown	
	PA0394	*yggS* - conserved hypothetical protein (4)		
S2A4	**PA1001**^e^	*phnA* - anthranilate synthase component I (1)	Adaptation, protection; amino acid biosynthesis	
	**PA1002**^e^	*phnB* - anthranilate synthase component II (1)		

^a^Inserts with antisense orientation are in bold.

^b^Loci included in the insert are in bold.

^c^Annotations according to the Pseudomonas Genome Database (http://www.pseudomonas.com) [27]. Numbers inside parenthesis indicate the classes of product name confidence. Class1: Function experimentally demonstrated in *P. aeruginosa*; Class 2: Function of highly similar gene experimentally demonstrated in another organism; Class 3: Function proposed based on presence of conserved amino acid motif, structural feature or limited sequence similarity to an experimentally studied gene. Class 4: Homologs of previously reported genes of unknown function, or no similarity to any previously reported sequences.

^d^DEG: Database of Essential Genes (DEG 7.0) (http://www.essentialgene.org) [20].

^e^Previous reports [34,35] did not mention growth defects associated to deletion of *phnAB* genes.

## Discussion

The discovery of novel essential genes or pathways that have not yet been targeted by clinical antibiotics can underlie the development of alternative effective antibacterials to overcome the extant mechanisms of resistance. In *P. aeruginosa*, a genome-wide assessment of essential genes has been performed previously by constructing an ordered, nonredundant random transposon (Tn) insertion library [9,10,23]. An approach of this kind has proven invaluable in studying bacterial genomes and in detecting novel essential genes. However, there can be some degree of imprecision in tagging for essentiality owing to Tn insertions into possible permissive site(s) of essential genes. For example, “classical” essential genes involved in DNA replication, transcription, translation, and cell division (e.g. *polA*, *holE*, *holB*, *holC, dnaG*, *dnaJ*, *dnaK*, *rpoC*, *infC*, and *ftsYEX*) were Tn inserted in previous investigations (Additional file 4: Table S4) [9,10,23] and, for this reason, *P. aeruginosa* alleles were not included in the Database of Essential Genes (DEG) [20]. Some disadvantages of this kind of approach could be overcome by using growth-conditional mutagenesis.

To generate growth-conditional phenotypes, we decided to use the antisense-mediated strategy established previously in *S. aureus*[13,14]. This technique is not affected by some of the bias linked to transposon mutagenesis mentioned above. However, it can present limitations in the multi-step process of antisense libraries preparation such as the blunt-end cloning of mechanically sheared DNA fragments, library clones carrying multigenic inserts, the reintroduction efficiency of libraries into the original host. In addition, the efficiency of antisense inhibition, supposed to affect gene translatability and/or mRNA stability, can be gene-dependent and also differential for distinct DNA fragments belonging to the same gene.

We report here, for the first time, successful application of regulated antisense RNA technology to discover novel essential functions in *P. aeruginosa*. To also screen for low expressed essential genes, we added a preliminary shotgun library construction in *E. coli* to the previous strategy, followed by mating transfer to *P. aeruginosa*.

The subset of growth-impairing fragments that targeted single loci (Table 1) directly defined 28 “essential-for-growth” genes. Only five of these genes were “classical” essential genes involved in DNA replication, transcription, and translation. The remaining 23 genes are suggested to take part in disparate cellular functions, including protein secretion, biosynthesis of cofactors, prosthetic groups, and carriers, energy metabolism, central intermediary metabolism, transport of small molecules, translation, post-translational modification, non-ribosomal peptide synthesis, lipopolysaccharide synthesis/modification, and transcriptional regulation. Finally, some of the gene products described in Table 1 were annotated as “hypothetical” proteins. We suggest that these proteins may be involved in unexplored essential functions, either as stand-alone proteins or connected to classical housekeeping processes. This is the case for the inner membrane protein TgpA (PA2873; Table 1) [21], which was found in our antisense screenings and was previously reported as hypothetical, whose transglutaminase activity associated with the periplasmic domain might be either linked to cell wall metabolism or be involved in unknown key functions of protein maturation, secretion, and/or modification.

Only two of the 23 non-classical essential genes, PA4669 (*ipk*) and PA3820 (*secF*), were already indicated as essential in *P. aeruginosa*[9,20]. For the remaining 21 genes, no evidence for essentiality has been reported previously in *P. aeruginosa*[20]. We propose these genes as novel essential genes in *P. aeruginosa*. PA2951 (*etfA*), PA3687 (*ppc*), PA3758 (*nagA*), PA1183 (*dctA*), and PA1805 (*ppiD*) are homologous to genes previously shown to be essential in a limited number of bacterial species [20]. Interestingly, for the remaining 16 genes, no homologs have been reported as essential in other bacteria [20]. Among these, PA1709 (*popD*), coding for a subunit of the PopB/D translocon complex of the type III secretion-translocation system (TTSS), is implicated in effector translocation across the host plasma membrane. Previous reports on *P. aeruginosa* PopD function [24-26] did not mention growth defects associated to deletion of *popD* gene. Therefore, the growth-impairing effects of S5A10 insert corresponding to PA1709 (Table 1) did not seem to match the PopD role characterized so far. These discrepancies could be due to differences in experimental conditions between our study and earlier works.

We evaluated the set of 21 novel candidate essential genes for degree of conservation in *Pseudomonas* species according to the computationally-based analysis of orthologs of the Pseudomonas Genome Database [27] (Additional file 5: Table S5). Interestingly, they are well-conserved in the sequenced *Pseudomonas* species, with the exceptions of PA5548 and PA1709 (*popD*) that are unique in *P. aeruginosa*. However, PA5548 and PA1709 (*popD*) orthologs can be found in other bacterial species. Remarkably, 17 of 21 novel essential candidates are conserved in all twelve sequenced *P. aeruginosa* genomes (Additional file 5: Table S5). Instead, PA2220 (*oprR*), PA5264, PA1709 (*popD*) and PA3687 (*ppc*) are present in 3, 8, 9 and 10 of the sequenced genomes, respectively. Essential genes that are not fully conserved in all strains of a bacterial species can occur infrequently. As an example, the *Escherichia coli* genes *ytfI, ypjF, ymfJ, ymfI* and *ymcD*, coding for hypothetical proteins, were reported as essential in the K12-MG1655 strain [28,29] and are conserved in only a limited number of the sequenced *E. coli* genomes [30].

Moreover, we compared the novel essential candidates with a panel of “classical” essential genes that were not included in the Database of Essential Genes (DEG) [20] because of the occurence of Tn insertions in previous screenings in *P. aeruginosa*[9,10,23]. The Tn insertion patterns of the novel essential candidates (i.e. number of insertions and insertion site(s)- terminal *vs* internal; Additional file 5: Table S5) were similar to those of “classical” essential genes (Additional file 4: Table S4).

This study also identified growth-impairing inserts carrying multiple genes. Because of their multigenic composition, the tagging of genes in these constructs for essentiality is not as direct as for single locus inserts (see above). However, among the multigenic inserts, we identified sequences corresponding to 25 genes with homologs already annotated as essential in other bacterial species [20] (Additional file 3: Table S3). Seven of these genes were indicated previously as essential in *P. aeruginosa*[9,20]. The 25 genes were annotated as involved in multiple cellular functions: lipid A biosynthesis (*lpxA, lpxB*; *lpxD*, *fabZ*) [31], amino acid biosynthesis and metabolism (*glyA3, proC, hom, lysC, ldh*), DNA replication and recombination (*dnaX, recB, recR*), transport of small molecules (*potD*, *mgtA, fadL, fepG*, *pstC)*, biosynthesis of cofactors, prosthetic groups and carriers (*folD*), translation and post-translational modification (*tufB*), nucleotide biosynthesis (*purL*), protein secretion (*secE*), tRNA modification (*gcp*) [32], central intermediary metabolism (*glpK),* and energy metabolism (*fdx2*). Other genes present in the multigenic inserts might be essential, but their identification would require further analysis via subcloning and/or conditional mutagenesis.

Interestingly, four multigenic inserts contained genes belonging to a single operon (Table 2), a feature that suggests a functional association. One such gene, *proC*, codes for pyrroline-5-carboxylate reductase [33] and was reported as essential in *E. coli*, *Mycobacterium tuberculosis* and *Acinetobacter baylyi*[20]. Other gene products of these operons are annotated as hypothetical proteins. Therefore, we suggest that these operonic genes might be involved in novel essential pathways. Overall, they are well-conserved in the sequenced *Pseudomonas* species (Additional file 5: Table S5). Exceptions are PA1088-1089-1090 which appear restricted to few *Pseudomonas* species and not conserved in all sequenced *P. aeruginosa* strains. Finally, one operonic growth-impairing insert included PA1001-1002 (*phnAB*) implicated in the biosynthesis of pyocyanin. Previous reports on *P. aeruginosa* PAO1 *phnA* and PA14 *phnAB* function [34,35] did not mention growth defects associated to deletion of these genes. As in the case of PA1709 (*popD*), discrepancies between our results and previous works could be attributable to differences in experimental conditions.

## Conclusions

Taken together, our results show the feasibility of antisense technology in *P. aeruginosa* for identifying novel essential genes. Because of its supposed inefficiency [16], this approach has been neglected in Gram-negative bacteria for several years, and was only recently recovered in *E. coli*[17]. By comparison with this previous work, the results presented here strongly suggest that our modification of the antisense strategy could broaden the class variety of the identified essential genes. We expect that our methodology could be well suited for antisense-mediated searches of essential genes in other Gram-negative bacterial species.

## Methods

### Bacterial strains, plasmids, and growth conditions

Bacterial strains and plasmids used in this study are listed in Additional file 6: Table S1. Bacteria were grown at 37°C in Luria-Bertani (LB) broth, or in M9 minimal medium supplemented with 0.2% citrate (M9-citrate). Antibiotics were added at the following concentrations (μg/ml): Cb, 300; kanamycin; 50. Arabinose was added to a final concentration of 10 mM. In mating experiments, exconjugant *P. aeruginosa* PAO1 clones were selected on PIA (Difco) containing Cb.

### Construction and screening of PAO1 shotgun antisense libraries

Genomic DNA was isolated from *P. aeruginosa* PAO1 using an illustra GenomicPrep Cells and Tissue DNA Isolation Kit (GE Healthcare). DNA was diluted in 10 mM TE buffer (pH 8.0) and nebulized to obtain sheared fragments spanning 200–800 bp (Additional file 1: Figure S1A). Following ethanol precipitation, fragmented DNA was treated with nuclease BAL-31 and Klenow (New England Biolabs) for 10 min at 30°C to obtain blunt ends. After enzyme inactivation with 1 mM EDTA, DNA was dialyzed against 20 mM Tris–HCl (pH 8.0). pVI533EH and pHERD20T were digested with *Sma*I (New England Biolabs) and dephosphorylated using shrimp alkaline phosphatase (Roche). Fragmented DNA was ligated to dephosphorylated vectors using T4 Ligase (Takara Bio) at 16°C overnight. Ligation mixtures were transformed into *E. coli* JM109 by electroporation, and transformants were selected on LB plates supplemented with Cb. The resulting transformant colonies composing the SAL were arrayed and cultured in 96-well microplates. Quality control by PCR of single colonies, using primers flanking the multi-cloning site (Additional file 1: Figure S1B), was performed to check the presence and the size of a genomic insert.

SALs were mobilized from *E. coli* to *P. aeruginosa* PAO1 by conjugative triparental mating. *E. coli* donor strains were grown overnight in 96-well microplates in LB broth supplemented with Cb. The recipient *P. aeruginosa* PAO1 and helper *E. coli* HB101/pRK2013 strains were grown overnight in flasks in LB broth. Thirty microliters each of helper, recipient, and donor strains were mixed in microplate wells. After mixing, microplates were centrifuged at 750 × g for 5 min and incubated for 3 h at 37°C. Cell pellets resulting from triparental mating were resuspended in 90 μl of LB, and 2 μl of each mating mixture were spotted on PIA plates supplemented with Cb, both in the absence and presence of 10 mM arabinose, to counter select *E. coli* donor and helper strains. Exconjugant cell spots were inspected for growth defects following 24–48 h of incubation at 37°C. The PAO1 growth-impairing inserts in pVI533EH/pHERD20T derivatives were sequenced following PCR amplification using oligo pVI533-F/pVI533-R and pHERD-F/pHERD-R, respectively (Additional file 6: Table S1). The resulting sequences were matched to the PAO1 genome at the *Pseudomonas* Genome Database [27].

## Authors’ contributions

RR, DV, FV, and GB conceived and designed the experiments. RR, RM, and FV performed the experiments. RR, DV, and GB analyzed the data. DV and GB wrote the paper. All authors read and approved the final manuscript.

## Supplementary Material

Additional file 1: Figure S1 Construction and screening for growth defects of *P. aeruginosa* shotgun antisense libraries. A. Agarose gel electrophoresis showing two fractions, F1 and F2 (lanes 2 and 3), of DNA fragments generated from *P. aeruginosa* PAO1 genomic DNA (lane 1). The DNA fragments from F1 and F2 were generated by nebulization at 2.5 and 5 bar pressure, respectively. B. Quality control for cloning: pHERD vector used for library preparation allows white/blue screening for positive inserts. White clones were checked by PCR for the presence of an insert using oligos annealing at both sides of the polylinker sequence. As an example, a check of a randomly selected pool of 25 white colonies is shown (M: molecular weight marker; E. empty vector). It is noteworthy that more than 90% of clones from F1 (23/25) carried an insert within the expected size range (200–800 bp; average size: 500 bp), and were used for shotgun cloning. C. SAL recipient PAO1 exconjugants were selected by spotting on PIA plates supplemented with Cb, both in the absence and in the presence of the P_BAD_ inducer arabinose. Recipient PAO1 exconjugant spots were inspected for growth defects following 24 h of incubation at 37°C. For example: red circle indicates growth impairment only with inducer; yellow circle indicates lethal effects only with inducer; green circle indicates lethal effects both in the presence and absence of the inducer. The identity of the genomic fragments eliciting growth was determined by sequencing the inserts in the corresponding clones of *E. coli* SAL. Click here for file

Additional file 2: Table S2 Growth-impairing inserts resulting from PAO1 SAL screenings. Click here for file

Additional file 3: Table S3 PAO1 growth-impairing inserts including multiple loci. Click here for file

Additional file 4: Table S4 Additional information on a selection of PAO1 “classical” essential genes. Click here for file

Additional file 5: Table S5 Additional information on novel *P. aeruginosa* candidate essential genes. Click here for file

Additional file 6: Table S1 List of bacterial strains, plasmids, and oligonucleotides. Click here for file
