# Supplementary figures and images for: A shotgun antisense approach to the identification of novel essential genes in Pseudomonas aeruginosa
Source: BMC Microbiol. 2014 Feb 5;14:24. doi: 10.1186/1471-2180-14-24 (PMC3922391; doi:10.1186/1471-2180-14-24)

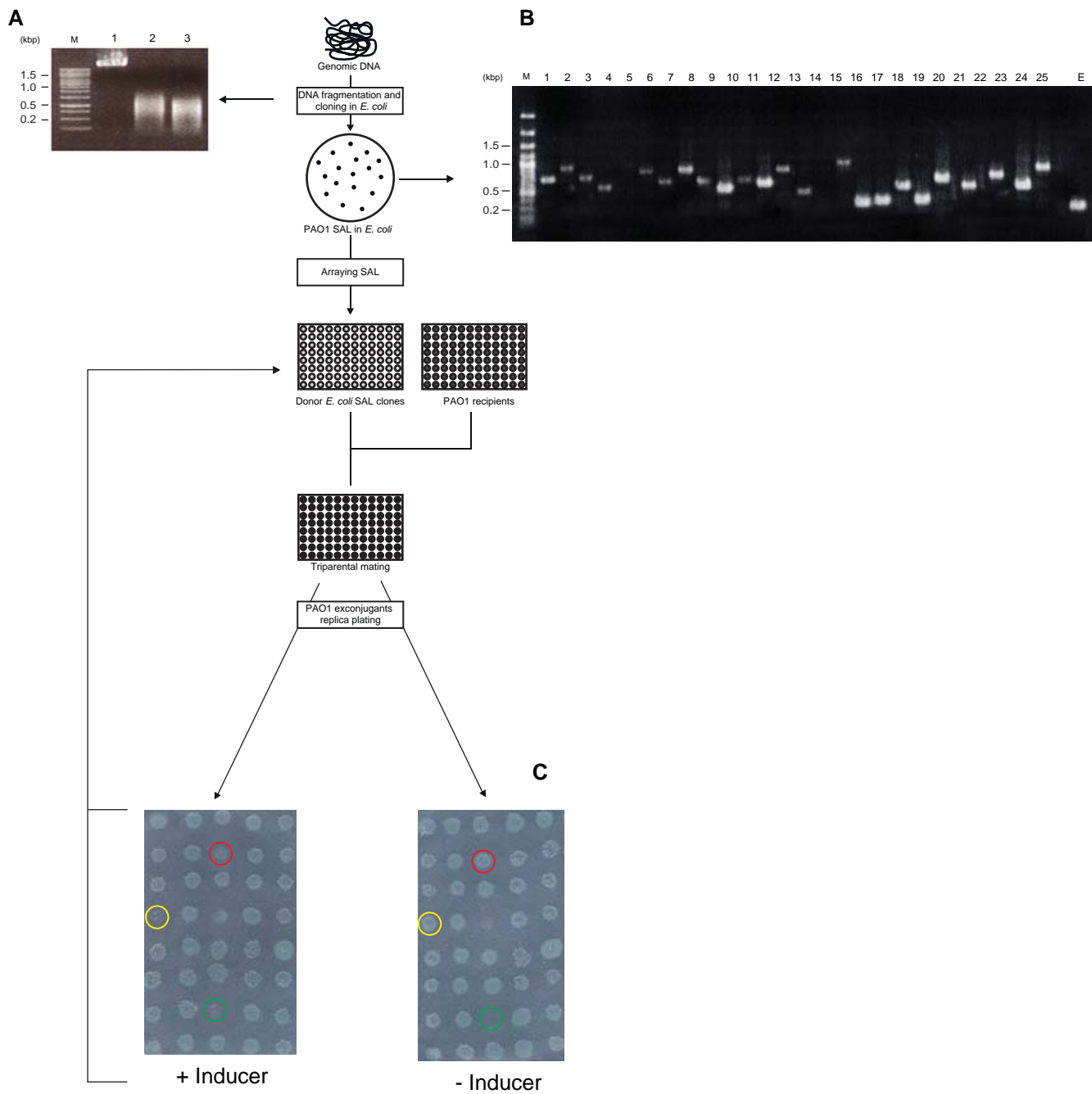

Supplement: Additional file 1: Figure S1 — Construction and screening for growth defects of P. aeruginosa shotgun antisense libraries. A. Agarose gel electrophoresis showing two fractions, F1 and F2 (lanes 2 and 3), of DNA fragments generated from P. aeruginosa PAO1 genomic DNA (lane 1). The DNA fragments from F1 and F2 were generated by nebulization at 2.5 and 5 bar pressure, respectively. B. Quality control for cloning: pHERD vector used for library preparation allows white/blue screening for positive inserts. White clones were checked by PCR for the presence of an insert using oligos annealing at both sides of the polylinker sequence. As an example, a check of a randomly selected pool of 25 white colonies is shown (M: molecular weight marker; E. empty vector). It is noteworthy that more than 90% of clones from F1 (23/25) carried an insert within the expected size range (200–800 bp; average size: 500 bp), and were used for shotgun cloning. C. SAL recipient PAO1 exconjugants were selected by spotting on PIA plates supplemented with Cb, both in the absence and in the presence of the PBAD inducer arabinose. Recipient PAO1 exconjugant spots were inspected for growth defects following 24 h of incubation at 37°C. For example: red circle indicates growth impairment only with inducer; yellow circle indicates lethal effects only with inducer; green circle indicates lethal effects both in the presence and absence of the inducer. The identity of the genomic fragments eliciting growth was determined by sequencing the inserts in the corresponding clones of E. coli SAL. [file 1471-2180-14-24-S1.pdf]
