# Supplementary material for: A shotgun antisense approach to the identification of novel essential genes in Pseudomonas aeruginosa
Source: BMC Microbiol. 2014 Feb 5;14:24. doi: 10.1186/1471-2180-14-24 (PMC3922391; doi:10.1186/1471-2180-14-24)
Supplement: Additional file 2: Table S2 — Growth-impairing inserts resulting from PAO1 SAL screenings. [file 1471-2180-14-24-S2.pdf]

Table S2. Growth-impairing inserts resulting from PAO1 SAL screenings.

Insert name[a]	Phenotype[b]		Start[c]	Stop[c]	Length (bp)	Orientation[d]	*Locus*[e]	Gene name and product annotation[f]
	+ ARA	- ARA						
**M4G6**	Lethal	Lethal	3226136	3226491	355	ANTISENSE	PA2873	tgpA - transglutaminase protein A TgpA (Class 1)
**M7E2**	Lethal	Defect	4443964	4443811	153	ANTISENSE	PA3964	hypothetical protein (Class 4)
			4443854	4443417	437	ANTISENSE	PA3963	yiiP - probable transporter (Class 3)
			596188	595707	481	SENSE	PA0536	hypothetical protein (Class 4)
**M2D2**	Defect	Normal	5121704	5121898	194		IR	
			5121899	5122199	300	ANTISENSE	PA4574	conserved hypothetical protein (Class 4)
			2651256	2650985	271	ANTISENSE	PA2394	pvdN (Class 1)
**M7G4**	Lethal	Defect	5950869	5951115	246		IR	
			5951115	5951612	497	ANTISENSE	PA5287	amtB - ammonium transporter AmtB (Class 2)
			4189311	4189547	236		IR	
			4189547	4189730	183	ANTISENSE	PA3736	hom - homoserine dehydrogenase (Class 1)
			3467036	3466960	76		IR	
			3466960	3466730	230	SENSE	PA3088	conserved hypothetical protein (Class 4)
**M7H8**	Defect	Normal	1953080	1952665	415	ANTISENSE	PA1800	tig - trigger factor (Class 2)
			1952665	1952433	232		IR	
			1952433	1952250	183	SENSE	PA1799	parR - two-component response regulator, ParR (Class 1)
A1	Lethal	Defect	5720756	5720945	189	SENSE	PA5081	hypothetical protein (Class 4)
			5720950	5721501	551	ANTISENSE	PA5082	probable binding protein component of ABC transporter (Class 3)
			5159950	5160615	665	SENSE	PA4601	morA - motility regulator (Class 1)
			5160616	5160737	121		IR	
			5160738	5160846	108	SENSE	PA4602	glyA3 - serine hydroxymethyltransferase (Class 2)
A5	Defect	Normal	1733430	1733642	212	SENSE	PA1590	braB - branched chain amino acid transporter (Class 1)
B1	Lethal	Defect	4778201	4778555	354	ANTISENSE	PA4270	rpoB - DNA-directed RNA polymerase beta chain (Class 2)
B3	Defect	Normal	6242757	6242573	184	ANTISENSE	PA5548	probable major facilitator superfamily (MFS) transporter (Class 3)
B5	Lethal	Defect	2124389	2124637	248	ANTISENSE	PA1941	hypothetical protein (Class 4)
			1946604	1946417	187	SENSE	PA1796	folD - 5,10-methylene-tetrahydrofolate dehydrogenase / cyclohydrolase (Class 2)
D1	Defect	Normal	6237168	6237657	489	ANTISENSE	PA5544	conserved hypothetical protein (Class 4)
			4044648	4044984	336	SENSE	PA3610	potD - polyamine transport protein PotD (Class 2)
			4044984	4045152	168		IR	
D2	Defect	Normal	1114549	1114297	252	ANTISENSE	PA1027	probable aldehyde dehydrogenase (Class 3)
			4267513	4267432	81	SENSE	PA3809	fdx2 - ferredoxin [2Fe-2S] (Class 2)
D5	Defect	Normal	2048624	2048570	54	SENSE	PA1878	hypothetical protein (Class 4)
			2048569	2048463	106		IR	
			2048462	2048258	204	ANTISENSE	PA1877	probable secretion protein (Class 3)
			6162859	6163204	345	SENSE	PA5473	conserved hypothetical protein (Class 4)
E2	Lethal	Defect	640687	640420	267	SENSE	PA0581	ygiH - conserved hypothetical protein (Class 4)
			640419	640342	77		IR	
			640341	640687	346	ANTISENSE	PA0580	gcp\ygjD O-sialoglycoprotein endopeptidase (Class 2)
E4	Defect	Normal	5138686	5138754	68	ANTISENSE	PA4588	gdhA - glutamate dehydrogenase (Class 2)

			5138755	5139049	294		IR	
			5139050	5139121	71	ANTISENSE	PA4589	fadL - probable outer membrane protein precursor (Class 3)
			3636174	3636093	81	SENSE	PA3249	probable transcriptional regulator (Class 3)
E4	Defect	Normal	5138686	5138754	68	ANTISENSE	PA4588	gdhA - glutamate dehydrogenase (Class 2)
			5138755	5139049	294		IR	
			5139050	5139121	71	ANTISENSE	PA4589	fadL - probable outer membrane protein precursor (Class 3)
			3636174	3636093	81	SENSE	PA3249	probable transcriptional regulator (Class 3)
E5	Lethal	Lethal	1285145	1284513	632	ANTISENSE	PA1183	dctA - C4-dicarboxylate transport protein (Class 2)
E6	Lethal	Defect	1125143	1125465	322	ANTISENSE	PA1037	yicG - conserved hypothetical protein (Class 4)
			1125466	1125547	81		IR	
			1125548	1125868	320	ANTISENSE	PA1038	hypothetical protein (Class 4)
F1	Defect	Normal	1692754	1692568	186	SENSE	PA1554	ccoN1 \ fixN \ Cytochrome c oxidase, cbb3-type, CcoN subunit (Class 1)
F2	Defect	Normal	5926859	5926602	257	SENSE	PA5264	hypothetical protein (Class 4)
G1	Defect	Normal	5197806	5197881	75		IR	
			5197881	5198071	190	SENSE	PA4630	hypothetical protein (Class 4)
			2007983	2007807	176	ANTISENSE	PA1847	yhgI - conserved hypothetical protein (Class 4)
H2	Lethal	Defect	3310951	3311112	161	ANTISENSE	PA2951	etfA - electron transfer flavoprotein alpha-subunit (Class 2)
G2	Lethal	Defect	3310792	3311282	490	ANTISENSE	PA2951	etfA - electron transfer flavoprotein alpha-subunit (Class 2)
G4	Defect	Normal	5862829	5862260	569	SENSE	PA5207	probable phosphate transporter (Class 3)
			3826157	3826019	138	ANTISENSE	PA3419	hypothetical protein (Class 4)
			3826019	3825774	245		IR	
			3825773	3825719	54	SENSE	PA3418	ldh - leucine dehydrogenase (Class 2)
S2A4	Lethal	Lethal	1087451	1085564	93	SENSE	PA1001	phnA - anthranilate synthase component I (Class 1)
			1085542	1085603	61	SENSE	PA1002	phnB - anthranilate synthase component II (Class 1)
S2F1	Lethal	Defect	637810	638141	331	ANTISENSE	PA0577	dnaG - DNA primase (Class 2)
S2F6	Defect	Normal	5327423	5327324	99	ANTISENSE	PA4743	rbfA - ribosome-binding factor A (Class 2)
S3C3	Defect	Normal	4784032	4784139	107	ANTISENSE	PA4276	secE \ prlG - secretion protein SecE (Class 2)
			4784140	4784183	43		IR	
			4784184	4784259	75	ANTISENSE	PA4276.1	tRNA-Trp (Class 2)
			4784260	4784315	55		IR	
			4784316	4784670	354	ANTISENSE	PA4277	tufB - elongation factor Tu (Class 2)
S3D3	Lethal	Defect	637230	636304	926	SENSE	PA0577	dnaG - DNA primase (Class 2)
S3D4	Lethal	Defect	1960531	1960182	349	ANTISENSE	PA1805	ppiD - peptidyl-prolyl cis-trans isomerase D (Class 2)
S4B7	Defect	Normal	4524644	4524114	530	SENSE	PA4041	hypothetical protein (Class 4)
			3131111	3131159	48	ANTISENSE	PA2772	hypothetical protein (Class 4)
			3131160	3131448	288		IR	
S4B10	Lethal	Defect	3841063	3841483	420	SENSE	PA3433	ywbI - probable transcriptional regulator (Class 3)
S4D10	Lethal	Defect	4082574	4082961	387	ANTISENSE	PA3644	lpxA - UDP-N-acetylglucosamine acyltransferase (Class 2)
			4082958	4083398	440	ANTISENSE	PA3645	fabZ - sefA - (3R)-hydroxymyristoyl-[acyl carrier protein] dehydratase (Class 2)
			4083399	4083443	44		IR	
			4083444	4084133	689	ANTISENSE	PA3646	lpxD \ omsA \ firA - UDP-3-O-[3-hydroxylauroyl] glucosamine N-acyltransferase (Class 2)
S4E6	Defect	Lethal	5502636	5502339	297	SENSE	PA4903	vanK - probable major facilitator superfamily (MFS) transporter (Class 3)

S4H12	Defect	Lethal	6241851	6242650	799	SENSE	PA5548	probable major facilitator superfamily (MFS) transporter (Class 3)
S5A1	Defect	Normal	2443160	2442692	468		IR	
			2442691	2442652	39	ANTISENSE	PA2220	oprR - probable transcriptional regulator (Class 3)
S5A4	Defect	Normal	5898131	5898063	68	SENSE	PA5238	probable O-antigen acetylase (Class 3)
S5A10	Lethal	Normal	1855763	1855737	26		IR	
			1855736	1855297	439	ANTISENSE	PA1709	popD \ pepD - Translocator outer membrane protein PopD precursor (Class 1)
S5D4	Defect	Normal	2677220	2676516	704	SENSE	PA2402	probable non-ribosomal peptide synthetase (Class 3)
S5D11	Lethal	Normal	3122034	3122265	231	ANTISENSE	PA2762	hypothetical protein (Class 4)
			3122266	3122297	31		IR	
			1671373	1672034	661	SENSE	PA1532	dnaX - DNA polymerase subunits gamma and tau (Class 2)
			1672035	1672079	44		IR	
			1672080	1672406	326	SENSE	PA1533	conserved hypothetical protein (Class 4)
			1672407	1672484	77		IR	
			1672485	1672830	345	SENSE	PA1534	recR - recombination protein RecR (Class 2)
S5G6	Lethal	Lethal	5898063	5898131	68	ANTISENSE	PA5238	probable O-antigen acetylase (Class 3)
S6E7	Lethal	Defect	4276723	4276362	361	SENSE	PA3820	secF - secretion protein SecF (Class 2)
S6A10	Defect	Normal	5327427	5327562	135	ANTISENSE	PA4744	infB - translation initiation factor IF-2 (Class 2)
S86C	Lethal	Defect	4208263	4208429	167	SENSE	PA3758	nagA - probable N-acetylglucosamine-6-phosphate deacetylase (Class 3)
S817E	Lethal	Defect	507855	507631	224	ANTISENSE	PA0451	conserved hypothetical protein (Class 4)
			507630	507570	60		IR	
			4081062	4081045	17	SENSE	PA3643	lpxB \ pgsB - lipid A-disaccharide synthase (Class 2)
			4081045	4080947	98	SENSE	PA3644	lpxA - UDP-N-acetylglucosamine acyltransferase (Class 2)
S828F	Lethal	Defect	5439259	5439758	499	SENSE	PA4844	probable chemotaxis transducer (Class 3)
			5439759	5439771	12		IR	
			1237546	1237480	66	ANTISENSE	PA1145	probable transcriptional regulator
			1237551	1238001	450	ANTISENSE	PA1146	probable iron-containing alcohol dehydrogenase (Class 3)
S841F	Lethal	Defect	5525057	5525437	382	ANTISENSE	PA4926	conserved hypothetical protein (Class 4)
S82G	Defect	Normal	3081915	3081418	498	ANTISENSE	PA2727	hypothetical protein (Class 4)
			5200935	5201381	446	SENSE	PA4633	probable chemotaxis transducer (Class 3)
S9A9	Defect	Normal	294329	294798	469	ANTISENSE	PA0262	conserved hypothetical protein (Class 4)
S9B6a	Lethal	Defect	1176726	1176982	256	SENSE	PA1089	conserved hypothetical protein (Class 4)
			1176958	1177036	78	SENSE	PA1090	conserved hypothetical protein (Class 4)
S9B6b	Lethal	Defect	434830	434894	64	ANTISENSE	PA0393	proC - pyrroline-5-carboxylate reductase (Class 1)
			434669	434819	150	ANTISENSE	PA0392	yggT - conserved hypothetical protein (Class 4)
S9C8	Lethal	Defect	234467	234112	355	ANTISENSE	PA0205	probable permease of ABC transporter (Class 3)
			153848	153837	11		IR	
			153836	153717	119	ANTISENSE	PA0135	hypothetical protein (Class 4)
S9C9	Defect	Normal	6037340	6036772	568	SENSE	PA5368	pstC - membrane protein component of ABC phosphate transporter (Class 1)
			2100707	2100289	418	ANTISENSE	PA1923	hypothetical protein (Class 4)
S9C59	Defect	Normal	154366	153837	529		IR	
			153836	153717	119	ANTISENSE	PA0135	hypothetical protein (Class 4)
			4569651	4569624	27		IR	
			4569623	4569283	340	SENSE	PA4086	cupB1 - probable fimbrial subunit CupB1 (Class 2)

S9E5	Defect	Normal	4145666	4144939	727	SENSE	PA3703	wspF - probable methylesterase (Class 3)
			4144938	4144832	106			
			4144831	4144743	88	SENSE	PA3702	wspR - WspR (Class 1)
S9G5	Defect	Normal	2674616	2674704	88	ANTISENSE	PA2402	probable non-ribosomal peptide synthetase (Class 3)
S10A3	Lethal	Normal	348093	348439	346		IR	
			348545	348440	105	ANTISENSE	PA0307	hypothetical protein (Class 4)
S10C8	Lethal	Normal	587898	587417	481	SENSE	PA0528	probable transcriptional regulator (Class 3)
			4014406	4014039	367	ANTISENSE	PA3582	glpK [Glycerokinase] - glycerol kinase (Class 1)
SC10D4	Lethal	Normal	2601262	2601490	228	ANTISENSE	PA2354	probable transcriptional regulator (Class 3)
			3453439	3452908	531	SENSE	PA3079	hypothetical protein (Class 4)
S10E3	Lethal	Normal	4657107	4657418	311	SENSE	PA4161	ferric enterobactin transport protein FepG (Class 2)
			4657429	4657617	188	ANTISENSE	PA4162	probable short-chain dehydrogenase (Class 3)
S10F8	Defect	Normal	5838470	5838167	303	SENSE	PA5186	probable iron-containing alcohol dehydrogenase (Class 3)
S11D9	Lethal	Normal	4615670	4616010	340	SENSE	PA4127	hpcG | hpaH - 2-oxo-hept-3-ene-1,7-dioate hydratase (Class 2)
			4215993	4216467	474	ANTISENSE	PA3763	purL - phosphoribosylformylglycinamidine synthase (Class 2)
			3118114	3118193	79	ANTISENSE	PA2757	hypothetical protein (Class 4)
			3118194	3118269	75		IR	
			2593153	2592957	196	ANTISENSE	PA2346	conserved hypothetical protein (Class 4)
S11E4	Lethal	Normal	1642573	1642998	425	ANTISENSE	PA1511	conserved hypothetical protein (Class 4)
			6093091	6093166	75		IR	
			6093167	6093317	150	ANTISENSE	PA5414	hypothetical protein (Class 4)
			2835461	2835306	155	SENSE	PA2518	xylX -toluate 1,2-dioxygenase alpha subunit-17 (Class 2)
S11F7	Lethal	Lethal	4775550	4775812	262	ANTISENSE	PA4269	rpoC-DNA-directed RNA polymerase beta* chain (Class 2)
S11F9a	Lethal	Normal	3453439	3452912	527	SENSE	PA3079	hypothetical protein (Class4)
			2601262	2601490	228	ANTISENSE	PA2354	probable transcriptional regulator (Class 3)
S11B4	Defect	Normal	3256405	3256611	206	ANTISENSE	PA2902	hypothetical protein (Class 4)
			3256612	3256680	68		IR	
			3256681	3256742	61	ANTISENSE	PA2903	precorrin-3 methylase CobJ (Class 2)
			3278160	3278060	100	ANTISENSE	PA2922	probable hydrolase (Class 3)
			5945438	5945933	495	SENSE	PA5281	yigB -probable hydrolase (Class 3)
			5945934	5945962	28		IR	
			5945963	5946045	82	ANTISENSE	PA5282	probable major facilitator superfamily (MFS) transporter (Class 3)
S11C6a	Lethal	Lethal	3453439	3452908	531	SENSE	PA3079	hypothetical protein (Class 4)
			2601262	2601490	228	ANTISENSE	PA2354	probable transcriptional regulator (Class 3)
S11C9	Lethal	Normal	3788559	3789144	585	ANTISENSE	PA3382	phosphonate transport protein PhnE-13 (Class 2)
S11B8	Lethal	Lethal	990798	990828	30	ANTISENSE	PA0904	lysC (ask,akaB) aspartate kinase alpha and beta chain (Class 2)
			990829	991012	183		IR	
			991013	991198	185	ANTISENSE	PA0905	rsmA (csrA) carbon storage regulator (Class 1)
			991199	991260	61		IR	
			991260	991350	90	ANTISENSE	PA0905.1	tRNA-Ser (Class 2)
			991351	991468	117		IR	
			991469	991477	8	ANTISENSE	PA0905.2	tRNA-Arg (Class 2)
			5417750	5417935	185	ANTISENSE	PA4825	mgtA -Mg(2+) transport ATPase, P-type 2 (Class 2)

			2717298	2717430	132	ANTISENSE	PA2424	pvdL (Class 1)
S11C13	Lethal	Lethal	6125830	6126057	227	ANTISENSE	PA5441	hypothetical protein(Class 4)
			4347794	4347698	96	ANTISENSE	PA3880	conserved hypothetical protein (Class 4)
S11B13	Lethal	Lethal	5238083	5237858	225	ANTISENSE	PA4669	ipk isopentenyl monophosphate kinase ychB (Class 2)
S11C5	Defect	Defect	5276309	5276735	426	SENSE	PA4697	hypothetical protein (Class 4)
			5276736	5276842	106		IR	
			5276843	5276989	146	ANTISENSE	PA4698	hypothetical protein (Class 4)
S11G10	Lethal	Normal	4129492	4129146	346	SENSE	PA3687	ppc - phosphoenolpyruvate carboxylase (Class 2)
S11E5	Lethal	Normal	2825463	2824722	741	SENSE	PA2507	catA - catechol 1,2-dioxygenase (Class 2)
			1556884	1556766	118	ANTISENSE	PA1429	probable cation-transporting P-type ATPase (Class 3)
S11F13	Defect	Normal	1622665	1622929	264	ANTISENSE	PA1494	conserved hypothetical protein (Class 4)
			5196570	5196456	114	ANTISENSE	PA4628	lysP - lysine-specific permease (Class 2)
S11F14	Lethal	Normal	1931144	1931474	330	ANTISENSE	PA1783	nasA - nasT nitrate transporter (Class 2)
			6074820	6074559	261	ANTISENSE	PA5396	hypothetical protein (Class 4)
			4803013	4803126	113	ANTISENSE	PA4284	recB - rorA - exodeoxyribonuclease V beta chain (Class 2)
			1295287	1295255	32	SENSE	PA1194	probable amino acid permease (Class 3)
S11D5	Defect	Normal	534945	534676	269	ANTISENSE	PA0473	psfA - probable glutathione S-transferase (Class 3)
			535085	534946	139		IR	
			535399	535086	313	ANTISENSE	PA0474	hypothetical protein (Class 4)

[a] Inserts resulting from the first pilot SAL screening are in bold.
[b] SAL recipient PAO1 exconjugants phenotype in presence (+ ARA) and absence (- ARA) of the P_{BAD} inducer.
[c] End coordinates of the insert according to the Pseudomonas Genome Database (www.pseudomonas.com) [1].
[d] Insert orientation relatively to the P_{BAD} of the cloning vector.
[e,f] PA number and annotations according to the Pseudomonas Genome Database (www.pseudomonas.com) [1]. IR: intergenic region.

1. Winsor GL, Lam DK, Fleming L, Lo R, Whiteside MD, Yu NY, Hancock RE, Brinkman FS: **Pseudomonas Genome Database: improved comparative analysis and population genomics capability for Pseudomonas genomes.** *Nucleic acids research* 2011, **39:**D596-600.
