# Supplementary material for: A shotgun antisense approach to the identification of novel essential genes in Pseudomonas aeruginosa
Source: BMC Microbiol. 2014 Feb 5;14:24. doi: 10.1186/1471-2180-14-24 (PMC3922391; doi:10.1186/1471-2180-14-24)
Supplement: Additional file 3: Table S3 — PAO1 growth-impairing inserts including multiple loci. [file 1471-2180-14-24-S3.pdf]

Table S3. PAO1 growth-impairing inserts including multiple *loci*

Insert name[a]	Insert-included *loci*[b]	Gene name and product annotation[c]	Species containing orthologs in DEG[d]
**S3C3**	PA4276	*secE \ prlG* - secretion protein SecE (Class 2)	Bs, Vc, Ec, Hi, Mg, Mt, Fn, Ab, Se, Sa, Ec, Ss
	PA4276.1	*tRNA-Trp* Rho-independent transcription terminator (Class 2)	
	PA4277	*tufB* - elongation factor Tu (Class 2)	Ec
**S4D10**	**PA3644**	*lpxA* - UDP-N-acetylglucosamine acyltransferase (Class 2)	Ec, Hp, St, Fn, Pa, Se,Ec
	PA3645	*fabZ* - sefA - (3R)-hydroxymyristoyl-[acyl carrier protein] dehydratase (Class 2)	Vc, Ec, Hi, Sp, Fn, Ab, Pa, Se, Sa, Ec, Cc, Ss
	PA3646	*lpxD \ omsA \ firA* - UDP-3-O-[3-hydroxylauroyl] glucosamine N-acyltransferase (Class 2)	Vc, Ec, Hi, Hp, Fn, Ab, Se, Cc, Pg
**E2**	PA0581	*ygiH* - conserved hypothetical protein (Class 4)	
	PA0580	*gcp\ygjD* O-sialoglycoprotein endopeptidase (Class 2)	Bs, Vc, Ec, Hi, Mg, Mt, Fn, Ab, Se, Sa, Ec, Ss
B5	PA1941	hypothetical protein (Class 4)	
	PA1796	*folD* - 5,10-methylene-tetrahydrofolate dehydrogenase (Class 2)	Bs, Ec, Mg, Sp, Mt, St, Fn, Ab, Mp, Pa, Se, Ec
A1	PA5081	hypothetical protein (Class 4)	
	PA5082	probable binding protein component of ABC transporter (Class 3)	
	PA4601	*morA* - motility regulator (Class 1)	
	PA4602	*glyA3* - serine hydroxymethyltransferase (Class 2)	Bs, Sa, Ec, Mg, Fn, Ab, Mp, Pa, Se, Ec
S817E	PA0451	conserved hypothetical protein (Class 4)	
	PA3643	*lpxB \ pgsB* - lipid A-disaccharide synthase (Class 2)	Vc, Ec, Fn, Ab, Pa, Se
	**PA3644**	*lpxA* - UDP-N-acetylglucosamine acyltransferase (Class 2)	Ec, Hp, St, Fn, Pa, Se,Ec
S5D11	PA2762	hypothetical protein (Class 4)	
	PA1532	*dnaX* - DNA polymerase subunits gamma and tau (Class 2)	Bs, Sa, Ec, Fn, Ab, Mp, Se, Ss
	PA1533	conserved hypothetical protein (Class 4)	
	PA1534	*recR* - recombination protein RecR (Class 2)	Pg
**S9B6b**[e]	PA0393	*proC* - pyrroline-5-carboxylate reductase (Class 1)	Ec, Mt, Ab,
	PA0392	*yggT* - conserved hypothetical protein (Class 4)	
D1	PA5544	conserved hypothetical protein (Class 4)	
	PA3610	*potD* - polyamine transport protein PotD (Class 2)	Ec, Hi, Mp
S11D9	PA4127	*hpcG* | hpaH - 2-oxo-hept-3-ene-1,7-dioate hydratase (Class 2)	
	PA3763	*purL* - phosphoribosylformylglycinamidine synthase (Class 2)	Hi, Mt, Ab
	PA2757	hypothetical protein (Class 4)	
	PA2346	conserved hypothetical protein (Class 3)	
S10C8	PA0528	probable transcriptional regulator (Class 3)	
	PA3582	*glpK* - glycerol kinase (Class 1)	Sa, Mg
M7G4	PA5287	*amtB* - ammonium transporter AmtB (Class 1)	
	PA3736	*hom* - homoserine dehydrogenase (Class 1)	Ab
	PA3088	conserved hypothetical protein (Class 3)	
S11B8	PA0904	*lysC* (*ask*,*akaB*) aspartate kinase alpha and beta chain (Class 2)	Hp, Ab
	PA0905	rsmA (csrA)carbon storage regulator (Class 1)	
	PA0905.1	tRNA-Ser	
	PA0905.2	tRNA-Arg	
	PA4825	*mgtA* -Mg(2+) transport ATPase, P-type 2 (Class 2)	Mp
	PA2424	*pvdL* (Class 1)	
E4	PA4588	*gdhA* - glutamate dehydrogenase (Class 2)	
	hPA4589	*fadL* - probable outer membrane protein precursor (Class 3)	St
	PA3249	probable transcriptional regulator (Class 3)	
G4	PA5207	probable phosphate transporter (Class 3)	

	PA3419	hypothetical protein (Class 4)	
	PA3418	*ldh* - leucine dehydrogenase (Class 2)	Hi
S9C9	PA5368	*pstC* - membrane protein component of ABC phosphate transporter (Class 1)	Ss
	PA1923	hypothetical protein (Class 4)	
**S10E3**	PA4161	ferric enterobactin transport protein FepG (Class 2)	Se
	PA4162	probable short-chain dehydrogenase (Class 3)	
D2	PA1027	probable aldehyde dehydrogenase (Class 3)-	
	PA3809	*fdx2* - ferredoxin [2Fe-2S] (Class 2)	Pa
S11F14	PA1783	*nasA* - nitrate transporter (Class 2)	
	PA5396	hypothetical protein (Class 4)	
	PA4284	*recB* - *rorA* - exodeoxyribonuclease V beta chain (Class 2)	Hi
	PA1194	probable amino acid permease (Class 3)	
**E6**[e]	PA1037	*yicG* - conserved hypothetical protein (Class 4)	
	PA1038	hypothetical protein (Class 4)	
**S2A4**[e]	PA1001	*phnA* - anthranilate synthase component I (Class 1)	
	PA1002	*phnB* - anthranilate synthase component II (Class 1)	
**S9B6a**[e]	PA1089	conserved hypothetical protein (Class 4)	
	PA1090	conserved hypothetical protein (Class 4)	
**M7H8**	PA1800	*tig* - trigger factor (Class 2)	
	PA1799	*parR* - two-component response regulator, ParR (Class 1)	
**S9E5**	PA3703	*wspF* - probable methylesterase (Class 3)	
	PA3702	*wspR* - WspR (Class 1)	
**S11C5**	PA4697	hypothetical protein (Class 4)	
	PA4698	hypothetical protein (Class 4)	
**S11D5**	PA0473	*psfA* - probable glutathione S-transferase (Class 3)	
	PA0474	hypothetical protein (Class 4)	
M7E2	PA3964	hypothetical protein (Class 4)	
	PA3963	*yiiP* - probable transporter (Class 3)	
	PA0536	hypothetical protein (Class 4)	
M2D2	PA4574	conserved hypothetical protein (Class 4)	
	PA2394	*pvdN* (Class 1)	
D5	PA1878	hypothetical protein (Class 4)	
	PA1877	probable secretion protein (Class 3)	
	PA5473	conserved hypothetical protein (Class 4)	
G1	PA4630	hypothetical protein (Class 4)	
	PA1847	*yhgI* - conserved hypothetical protein (Class 4)	
S4B7	PA4041	hypothetical protein (Class 4)	
	PA2772	hypothetical protein (Class 4)	
S828F	PA4844	probable chemotaxis transducer (Class 3)	
	PA1145	probable transcriptional regulator (Class 3)	
	PA1146	probable iron-containing alcohol dehydrogenase (Class 3)	
S82G	PA2727	hypothetical protein (Class 4)	
	PA4633	probable chemotaxis transducer (Class 3)	
S9C8	PA0205	probable permease of ABC transporter (Class 3)	
	**PA0135**	hypothetical protein (Class 4)	
S9C59	**PA0135**	hypothetical protein (Class 4)	
	PA4086	*cupB1* - probable fimbrial subunit CupB1 (Class 2)	
SC10D4	**PA2354**	probable transcriptional regulator (Class 3)	
	**PA3079**	hypothetical protein (Class 4)	

S11E4	PA1511	conserved hypothetical protein (Class 3)
	PA5414	hypothetical protein (Class 4)
	PA2518	*xylX* -toluate 1,2-dioxygenase alpha subunit (Class 2)
S11F9a	**PA3079**	hypothetical protein (Class4)
	**PA2354**	probable transcriptional regulator (Class 3)
S11B4	PA2902	hypothetical protein(Class4)
	PA2903	precorrin-3 methylase CobJ (Class 2)
	PA2922	probable hydrolase (Class 3)
	PA5281	*yigB* -probable hydrolase (Class 2)
	PA5282	probable major facilitator superfamily (MFS) transporter (Class 3)
S11C6a	**PA3079**	hypothetical protein (class 4)
	**PA2354**	probable transcriptional regulator (Class 3)
S11C13	PA5441	hypothetical protein(Class 4)
	PA3880	conserved hypothetical protein (Class 4)
S11E5	PA2507	*catA* - catechol 1,2-dioxygenase (Class 2)
	PA1429	probable cation-transporting P-type ATPase (Class 3)
S11F13	PA1494	conserved hypothetical protein (Class 3)
	PA4628	*lysP* - lysine-specific permease (Class 2)

[a] Non-chimeric inserts are in bold.
[b] *Loci* present in more than one insert are in bold.
[c] Annotations according to the Pseudomonas Genome Database (www.pseudomonas.com) [1].
[d] DEG: Database of Essential Genes (DEG 7.0) (www.essentialgene.org) [2]. Bacterial species: Ab (*Acinetobacter baylyi*), Bs (*Bacillus subtilis*), Bt (*Bacteroides thetaiotaomicron*), Cc (*Caulobacter crescentus*), Ec (*Escherichia coli*), Fn (*Francisella novicida*), Hf (*Haemophilus influenzae*), Hp (*Helicobacter pylori)*, Mt (*Mycobacterium tuberculosis*), Mp (*Mycoplasma pulmonis*), Mg (*Mycoplasma genitalium*), Pg (*Porphyromonas gingivalis*), Pa (*Pseudomonas aeruginosa*), Se (*Salmonella enterica*), St (*Salmonella typhimurium*), Sp (*Streptococcus pneumoniae*), Ss (*Streptococcus sanguinis*), Sa (S*taphylococcus aureus*), Vc (*Vibrio cholerae*).
[e] Inserts spanning adjacent ORFs belonging to an operon.

1. Winsor GL, Lam DK, Fleming L, Lo R, Whiteside MD, Yu NY, Hancock RE, Brinkman FS: **Pseudomonas Genome Database: improved comparative analysis and population genomics capability for Pseudomonas genomes.** *Nucleic acids research* 2011, **39:**D596-600.
2. Zhang R, Lin Y: **DEG 5.0, a database of essential genes in both prokaryotes and eukaryotes.** *Nucleic acids research* 2009, **37:**D455-458.
