# Supplementary material for: A shotgun antisense approach to the identification of novel essential genes in Pseudomonas aeruginosa
Source: BMC Microbiol. 2014 Feb 5;14:24. doi: 10.1186/1471-2180-14-24 (PMC3922391; doi:10.1186/1471-2180-14-24)
Supplement: Additional file 4: Table S4 — Additional information on a selection of PAO1 “classical” essential genes. [file 1471-2180-14-24-S4.pdf]

Table S4. Additional information on a selection of *P. aeruginosa* “classical” essential genes.

Locus	**Gene name and product annotation[a]**	**Putative orthologs in *Pseudomonas* species[b]**	**Transposon insertion (N[c], IP[d])**	**Notes**
PA0577	*dnaG* DNA primase	Pae(12/12), Pbr, Pde, Pen, Pfl, Pfu, Pme, Ppo, Ppr, Ppu, Pre, Pst, Psy	1(TA), 1992/1995; 1(TL), 1990/1995	PA14 ortholog was Tn inserted in [1]
PA5493	*polA* DNA polymerase I	Pae(10/12),Pbr, Pde, Pen, Pfl, Pfu, Pme, Ppo, Ppr, Ppu, Pre, Pst, Psy	2(TA), 2153/2742 - 1628/2742; 3(TL), 1158/2742 - 2328/2742 - 2309/2742	PA14 ortholog was Tn inserted in [1]
PA3832	*holC* DNA polymerase III, chi subunit	Pae(12/12),Pbr, Pde, Pen, Pfl, Pfu, Pme, Ppo, Ppr, Ppu, Pre, Pst, Psy		PA14 ortholog was Tn inserted in [1]
PA2961	*holB* DNA polymerase III, delta subunit	Pae(12/12) Pbr, Pde, Pen, Pfl, Pfu, Pme, Ppo, Ppr, Ppu, Pre, Pst, Psy	2(TA), 971/987 - 963/987; 2(TL), 953/987	PA14 ortholog was Tn inserted in [1]
PA4760	*dnaJ* protein	Pae(12/12), Pbr, Pde, Pen, Pfl, Pfu, Pme, Ppr, Ppu , Pre, Pst, Psy		PA14 ortholog was Tn inserted in [1]
PA4761	*dnaK* molecular chaperone DnaK	Pae(12/12), Pbr, Pde, Pen, Pfl, Pfu, Pme, Ppo, Ppr, Ppu, Pre, Pst, Psy		PA14 ortholog was Tn inserted in [1]
PA4269	*rpoC* RNA polymerase beta* chain	Pae(10/12), Pbr, Pde, Pen, Pfl, Pfu, Pme, Ppo, Ppr, Ppu, Pre, Pst, Psy		PA14 ortholog was Tn inserted in [1]
PA2473	*infC* Translation initiation factor IF-3	Pae(12/12), Pbr, Pde, Pen, Pfl, Pfu, Pme, Ppo, Ppr, Ppu, Pre, Pst, Psy		PA14 ortholog was Tn inserted in [1]
PA0373	*ftsY* cell division protein	Pae(12/12), Pbr, Pde, Pen, Pfl, Pfu, Pme, Ppo, Ppr, Ppu, Pre, Pst, Psy	1(TA), 1359/1368	PA14 ortholog was Tn inserted in [1]
PA0374	*ftsE* cell division protein	Pae(12/12), Pbr, Pde, Pen, Pfl, Pfu, Pme, Ppo, Ppr, Ppu, Pre, Pst, Psy	2(TA), 196/672 - 268/672	PA14 ortholog was Tn inserted in [1]
PA0375	*ftsX* cell division protein	Pae(12/12), Pbr, Pde, Pen, Pfl, Pfu, Pme, Ppo, Ppr, Ppu, Pre, Pst, Psy	1(TA), 36/1008; 1(TL) 548/1008	PA14 ortholog was Tn inserted in [1]

[a] Annotations according to the Pseudomonas Genome Database (www.pseudomonas.com)[2].

[b] For each hit, the computationally-predicted putative orthologs that were found in the Pseudomonas Genome Database (www.pseudomonas.com) [2] are indicated with the abbreviation of the harboring bacterial species. For *Pseudomonas aeruginosa*, the number of strains harboring orthologs over the total of 12 sequenced strains considered for the analysis at the Pseudomonas Genome Database is indicated inside parenthesis. Species name abbreviations: Pae (*P. aeruginosa*), Pen (*P. entomophila*), Pme (*P. mendocina*), Pre (*P. resinovorans*), Pfl (*P. fluorescens*), Pbr (*P. brassicacearum*), Ppr (*P. protegens*), Pde (*P. denitrificans*), Ppu (*P. putida*), Pfu (*P. fulva*), Pst (*P. stutzeri*), Psy (*P. syringae*), Ppo (*P. poae*).

[c] **N**: number of transposon insertions in the PA Two Allele Transposon Library (TA) [3] and/or in the Tn5 lux Transposon Mutant Library (TL) [4].

[d] **IP**: transposon insertion site given as number of base pairs from the gene 5’ / total gene length.

References

1. Liberati NT, Urbach JM, Miyata S, Lee DG, Drenkard E, Wu G, Villanueva J, Wei T, Ausubel FM: **An ordered, nonredundant library of *Pseudomonas aeruginosa* strain PA14 transposon insertion mutants.** *P Natl Acad Sci USA* 2006, **103:**2833-2838.
2. Winsor GL, Lam DK, Fleming L, Lo R, Whiteside MD, Yu NY, Hancock RE, Brinkman FS: **Pseudomonas Genome Database: improved comparative analysis and population genomics capability for *Pseudomonas* genomes.** *Nucleic acids research* 2011, **39:**D596-600.
3. Jacobs MA, Alwood A, Thaipisuttikul I, Spencer D, Haugen E, Ernst S, Will O, Kaul R, Raymond C, Levy R, et al: **Comprehensive transposon mutant library of *Pseudomonas aeruginosa*.** *Proc Natl Acad Sci U S A* 2003, **100:**14339-14344.
4. Lewenza S, Falsafi RK, Winsor G, Gooderham WJ, McPhee JB, Brinkman FS, Hancock RE: **Construction of a mini-Tn5-luxCDABE mutant library in *Pseudomonas aeruginosa* PAO1: a tool for identifying differentially regulated genes.** *Genome Res* 2005, **15:**583-589.
