# Supplementary material for: A shotgun antisense approach to the identification of novel essential genes in Pseudomonas aeruginosa
Source: BMC Microbiol. 2014 Feb 5;14:24. doi: 10.1186/1471-2180-14-24 (PMC3922391; doi:10.1186/1471-2180-14-24)
Supplement: Additional file 5: Table S5 — Additional information on novel P. aeruginosa candidate essential genes. [file 1471-2180-14-24-S5.pdf]

Table S5. Additional information on novel *P. aeruginosa* candidate essential genes.

Locus[a]	Gene name and product annotation[b]	Putative orthologous in *Pseudomonas* species[c]	Transposon insertion (N[d], IP[e])	Notes
PA2951	*etfA* - electron transfer flavoprotein alpha-subunit	Pae(12/12), Pbr, Pde, Pen, Pfl, Pfu, Pme, Ppo, Ppr, Ppu, Pre, Pst, Psy	0(TA) ; 0(TL)	PA2951 orthologs are reported as essential in *Acinetobacter baylyi*, *Caulobacter crescentus* [1].
PA3687	*ppc* - phosphoenolpyruvate carboxylase	Pae(10/12), Pbr, Pde, Pen, Pfl, Pfu, Pme, Ppo, Ppr, Ppu, Pre, Pst, Psy	2(TA), 2357/2637 - 1408/2637	PA3687 orthologs are reported as essential in *Haemophilus influenzae* [1]
PA3758	*nagA* - probable N-acetylglucosamine-6-phosphate deacetylase	Pae(12/12), Pbr, Pen, Pfl, Ppo, Ppr	2(TA), 552/1092 - 556/1092	PA3785 orthologs are reported as essential in *Haemophilus influenzae*, *Mycobacterium tuberculosis* [1]
PA1183	*dctA* - C4-dicarboxylate transport protein	Pae(12/12), Pbr, Pde, Pen, Pfl, Pfu, Ppo, Ppr, Ppu, Pre, Pst, Psy	2(TA), 286/1311 - 685/1311	PA1183 orthologs are reported as essential in *Acinetobacter baylyi* [1]
PA1805	*ppiD* - peptidyl-prolyl cis-trans isomerase D - Rotamase D	Pae(12/12), Pbr, Pde, Pen, Pfl, Pfu, Pme, Ppo, Ppr, Ppu, Pre, Pst, Psy	1(TA), 1429/1866	PA1805 orthologs are reported as essential in *Caulobacter crescentus*, *Bacillus subtilis* [1]
PA5186	probable iron-containing alcohol dehydrogenase	Pae(12/12), Pde, Pfu, Pme, Ppu, Pre, Pst	2(TA), 42/1164 - 797/1164; 1(TL), 524/1164	
PA1554	*ccoN1*\Cytochrome c oxidase, cbb3-type, CcoN subunit	Pae(12/12), Pbr, Pd, Pen, Pfl, Pfu, Pme, Ppo, Ppr, Ppu, Pre, Pst, Psy,	3(TA), 1188/1428 - 885/1428 - 419/1428	Shown to be critical for aerobic growth [2].
PA3382	*phnE* - phosphonate transport protein PhnE	Pae(12/12), Pde, Pfl, Pme, Ppu, Pst, Psy	3(TA), 661/795 - 450/795 - 298/795; 1(TL), 652/795	The phosphonate transporter function was reported as essential in *Mycoplasma pulmonis* [1].
PA4903	*vanK* - probable major facilitator superfamily transporter	Pae(12/12), Pde, Pfl, Pfu, Ppr, Ppu, Pre	1(TA), 713/1335	
PA5548	probable major facilitator superfamily transporter	Pae(12/12)	1(TA), 611/1206	
PA1709	*popD* - Translocator outer membrane protein	Pae(9/12)	1(TA), 871/888	
PA1590	*braB* - branched chain amino acid transporter	Pae(12/12), Pme, Pst	1(TA), 1080/1314	
PA2402	Probable non-ribosomal peptide synthetase	Pae(12/12), Pbr, Pde, Pen, Pfl, Pme, Ppo, Ppr, Ppu, Pre, Pst, Psy	2(TA), 10034/15450 - 462/15450	50% identity with the peptide synthase MbtE reported as essential in *Haemophilus influenzae* [1].
PA5238	probable O-antigen acetylase	Pae(12/12), Pbr, Pfl, Pfu, Pme, Ppo, Ppr, Ppu, Pre, Pst, Psy	2(TL), 915/1989 - 1698/1989	
PA3433	*ywbI* - probable transcriptional regulator	Pae(12/12), Pde, Pen, Pfl, Ppo, Ppr, Ppu, Pre, Psy	0(TA) ; 0(TL)	
PA2220	*oprR* - probable transcriptional regulator	Pae(3/12), Pbr, Pde, Pfl, Ppr, Ppu, Pst, Psy	1(TA), 58/921	
PA2873	*tgpA* - transglutaminase protein A TgpA	Pae(12/12), Pde, Pfl, Pme, Pre, Pst, Psy	1(TA), 1568/2007; 1(TL) 1042/2007	
PA0307	hypothetical protein	Pae(12/12), Pbr, Pde, Pen, Pfl, Pfu, Pme, Ppo, Ppr, Ppu, Pre, Pst	2(TA), 451/612 - 408/612	
PA4926	conserved hypothetical protein	Pae(12/12), Pbr, Pfl, Pfu, Pme, Ppo, Ppr, Pre, Psy	1(TA), 29/936	
PA0262	conserved hypothetical protein	Pae(12/12), Ppu, Pre, Psy	9(TL), 1703/3060 - 1223/3060 - 1214/3060 - 1203/3060 - 396/3060 - 1242/3060 - 1244/3060 - 1483/3060 - 1219/3060	
PA5264	hypothetical protein	Pae(8/12), Pde, Pst, Psy	2(TA), 567/972 - 559/972; 1(TL), 851/972	

Locus[a]	Gene name and product annotation[b]	Putative orthologous in *Pseudomonas* species[c]	Transposon insertion (N[d], IP[e])	Notes
**PA1037**	*yicG* - conserved hypothetical protein	Pae(12/12), Pbr, Pde, Pfl, Pen, Pfu, Pme, Ppo, Ppr, Ppu, Pre, Pst, Psy	2(TA), 331/621 - 85/621; 2(TL), 258/621 - 449/621	
**PA1038**	hypothetical protein	Pae(12/12)	2(TA), 109/318 - 82/318; 2(TL), 112/318 - 164/318	
**PA1039**	*ychJ* - hypotetical protein	Pae(12/12), Pbr, Pde, Pen, Pfl, Pfu, Pme, Ppo, Ppr, Ppu, Pre, Pst, Psy	1(TA), 230/474	
**PA1040**	hypothetical protein	Pae(12/12),Pbr, Pde, Pen, Pfl, Pfu, Pme, Ppo, Ppr, Ppu, Pre, Pst, Psy	1(TA), 494/498	
**PA1089**	conserved hypothetical protein	Pae(4/12), Pme	1(TA), 490/603	
**PA1090**	conserved hypothetical protein	Pae(4/12), Pme, Pfl	1(TA), 198/663	
**PA1088**	hypothetical protein	Pae(4/12), Pfu, Pme	1(TA), 37/762	
**PA0392**	*yggT* - conserved hypothetical protein	Pae(12/12),Pbr, Pde, Pen, Pfl, Pfu, Pme, Ppo, Ppr, Ppu, Pre, Pst, Psy	1(TA), 85/594	
**PA0393**	*proC* - pyrroline-5-carboxylate reductase	Pae(12/12),Pbr, Pde, Pen, Pfl, Pfu, Pme, Ppo, Ppr, Ppu, Pre, Pst, Psy	2(TA), 610/822 -414/822; 2(TL), 744/822 -743/822	PA0393 orthologs are reported as essential in *Escherichia coli*, *Mycobacterium tuberculosis*, *Acinetobacter baylyi* [1].
**PA0394**	*yggS* - conserved hypothetical protein	Pae(12/12),Pbr, Pde, Pen, Pfl, Pfu, Pme, Ppo, Ppr, Ppu, Pre, Pst, Psy	1(TA), 351/693	
**PA1001**	*phnA* - anthranilate synthase component I	Pae(12/12)		
**PA1002**	*phnB* - anthranilate synthase component I	Pae(12/12)	1(TA), 184/603	

[a] *Loci* from Table 2 belonging to a single operon are in bold.

[b] Annotations according to the Pseudomonas Genome Database (www.pseudomonas.com)[3].

[c] For each hit, the computationally-predicted putative orthologs that were found in the Pseudomonas Genome Database (www.pseudomonas.com) [3] are indicated with the abbreviation of the harboring bacterial species. For *Pseudomonas aeruginosa*, the number of strains harboring orthologs over the total of 12 sequenced strains considered for the analysis at the Pseudomonas Genome Database is indicated inside parenthesis. Species name abbreviations: Pae (*P. aeruginosa*), Pen (*P. entomophila*), Pme (*P. mendocina*), Pre (*P. resinovorans*), Pfl (*P. fluorescens*), Pbr (*P. brassicacearum*), Ppr (*P. protegens*), Pde (*P. denitrificans*), Ppu (*P. putida*), Pfu (*P. fulva*), Pst (*P. stutzeri*), Psy (*P. syringae*), Ppo (*P. poae*).

[d] **N**: number of transposon insertions in the PA Two Allele Transposon Library (TA) [4] and/or in the Tn5 lux Transposon Mutant Library (TL) [5]. For all hits, a PA14 ortholog was Tn inserted in [6].

[e] **IP**: transposon insertion site given as number of base pairs from the gene 5’ / total gene length.

References

1. Zhang R, Lin Y: **DEG 5.0, a database of essential genes in both prokaryotes and eukaryotes.** *Nucleic acids research* 2009, **37:**D455-458.
2. Comolli JC, Donohue TJ: **Differences in two *Pseudomonas aeruginosa* cbb3 cytochrome oxidases.** *Molecular microbiology* 2004, **51:**1193-1203.
3. Winsor GL, Lam DK, Fleming L, Lo R, Whiteside MD, Yu NY, Hancock RE, Brinkman FS: **Pseudomonas Genome Database: improved comparative analysis and population genomics capability for *Pseudomonas* genomes.** *Nucleic acids research* 2011, **39:**D596-600.
4. Jacobs MA, Alwood A, Thaipisuttikul I, Spencer D, Haugen E, Ernst S, Will O, Kaul R, Raymond C, Levy R, et al: **Comprehensive transposon mutant library of *Pseudomonas aeruginosa*.** *Proc Natl Acad Sci U S A* 2003, **100:**14339-14344.
5. Lewenza S, Falsafi RK, Winsor G, Gooderham WJ, McPhee JB, Brinkman FS, Hancock RE: **Construction of a mini-Tn5-luxCDABE mutant library in *Pseudomonas aeruginosa* PAO1: a tool for identifying differentially regulated genes.** *Genome Res* 2005, **15:**583-589.
6. Liberati NT, Urbach JM, Miyata S, Lee DG, Drenkard E, Wu G, Villanueva J, Wei T, Ausubel FM: **An ordered, nonredundant library of *Pseudomonas aeruginosa* strain PA14 transposon insertion mutants.** *P Natl Acad Sci USA* 2006, **103:**2833-2838.
