# Supplementary material for: A shotgun antisense approach to the identification of novel essential genes in Pseudomonas aeruginosa
Source: BMC Microbiol. 2014 Feb 5;14:24. doi: 10.1186/1471-2180-14-24 (PMC3922391; doi:10.1186/1471-2180-14-24)
Supplement: Additional file 6: Table S1 — List of bacterial strains, plasmids, and oligonucleotides. [file 1471-2180-14-24-S6.pdf]

Table S1: List of bacterial strains, plasmids and oligonucleotides.

Strains,plasmids,oligos	Genotype or description	Reference
Pseudomonas aeruginosa		
PAO1		[1]
Escherichia coli		
JM109	*recA1 endA1 gyrA96 thi hsdR17 supE44, relA1, Δ(lac-proAB)* F' *(traD36, proAB+ lacIqZ* ΔM15)	[2]
HB101 [RK2013]	*recA, thi, hsdR, hsdM, proA, leu, strA*	[3]
Plasmids		
pHERD20T	Ap^R, Cb^R broad-host-range *araC-P_{BAD}* expression vector; blue/white screening for recombinants	[4]
pVI533EH	Ap^R, Cb^R broad-host-range *araC-P_{BAD}*	[5]
Oligonucleotides		
pVI533-F	ATCACGGCAGAAAAGTCCAC	
pVI533-R	CTTCTCTCATCCGCCAAAAC	
E2Eco5'	CGCGAATTCTCAGACCGCCGGCAGCGAC	
E2Hind3'	CGCAAGCTTCTGGCAGCGTTGCGTCGA	
pHERD-F	ATCGCAACTCTCTACTGTTTCT	
pHERD-R	TGCAAGGCGATTAAGTTGGGT	

References

1. Stover CK, Pham XQ, Erwin AL, Mizoguchi SD, Warrener P, et al. (2000) Complete genome sequence of *Pseudomonas aeruginosa* PAO1, an opportunistic pathogen. Nature 406: 959-964.
2. Yanisch-Perron C, Vieira J, Messing J (1985) Improved M13 phage cloning vectors and host strains: nucleotide sequences of the M13mp18 and pUC19 vectors. 33: 103-119.
3. Boyer HW, Roulland-Dussoix D (1969) A complementation analysis of the restriction and modification of DNA in *Escherichia coli*. 41: 459-472.
4. Qiu D, Damron FH, Mima T, Schweizer HP, Yu HD (2008) PBAD-based shuttle vectors for functional analysis of toxic and highly regulated genes in *Pseudomonas* and *Burkholderia* spp. and other bacteria. 74: 7422-7426.
5. Sze CC, Shingler V (1999) The alarmone (p)ppGpp mediates physiological-responsive control at the sigma 54-dependent Po promoter. 31: 1217-1228.
